# The Effects of Electronic Health Records on Medical Error Reduction: Extension of the DeLone and McLean Information System Success Model

**DOI:** 10.2196/54572

**Published:** 2024-10-16

**Authors:** Bester Chimbo, Lovemore Motsi

**Affiliations:** 1 Department of Information Systems University of South Africa Johannesburg South Africa

**Keywords:** medication error, patient safety, information system, information systems, electronic health record, service quality

## Abstract

**Background:**

Medical errors are becoming a major problem for health care providers and those who design health policies. These errors cause patients’ illnesses to worsen over time and can make recovery impossible. For the benefit of patients and the welfare of health care providers, a decrease in these errors is required to maintain safe, high-quality patient care.

**Objective:**

This study aimed to improve the ability of health care professionals to diagnose diseases and reduce medical errors.

**Methods:**

Data collection was performed at Dr George Mukhari Academic Hospital using convenience sampling. In total, 300 health care professionals were given a self-administered questionnaire, including doctors, dentists, pharmacists, physiologists, and nurses. To test the study hypotheses, multiple linear regression was used to evaluate empirical data.

**Results:**

In the sample of 300 health care professionals, no significant correlation was found between medical error reduction (MER) and knowledge quality (KQ) (β=.043, *P*=.48). A nonsignificant negative relationship existed between MER and information quality (IQ) (β=–.080, *P*=.19). However, a significant positive relationship was observed between MER and electronic health records (EHR; β=.125, 95% CI 0.005-0.245, *P*=.042).

**Conclusions:**

Increasing patient access to medical records for health care professionals may significantly improve patient health and well-being. The effectiveness of health care organizations’ operations can also be increased through better health information systems. To lower medical errors and enhance patient outcomes, policy makers should provide financing and support for EHR adoption as a top priority. Health care administrators should also concentrate on providing staff with the training they need to operate these systems efficiently. Empirical surveys in other public and private hospitals can be used to further test the validated survey instrument.

## Introduction

### Background

Worldwide, the delivery of health care has been altered and improved through health information technology. In health care systems, patient administration and management have been facilitated by health information technology. The electronic health record (EHR) system is frequently cited as a vital piece of health information technology to raise the standard of patient care [1]. In the early 1970s, computerized patient EHR were first used to collect, save, and display patient data [2,3]. The ordering of tests, consultations, electronic prescriptions, decision support systems, digital imaging, telemedicine, and other clinical service units can all be included in EHRs, while preserving patient privacy and confidentiality [2].

According to Tegegne et al [4], both high-income and resource-constrained nations have the implementation of the EHR system on their priority agenda. Implementing an EHR is necessary to improve clinical judgment, patient information security, and privacy [1]. The EHR is thought to have the following potential advantages for the health care system: safety, patient information organization, care coordination, communication, patient history, quick access to medical information, and effectiveness of care [5,6]. Evidence also shows that EHR can improve data quality by storing patient data and performing medical tasks [7]. Furthermore, current studies on health information system (HIS) success are generally limited to exploring the driving factors at the HIS adoption level. However, the adoption of an HIS is not indicative of implementation success, as the value and potential of an HIS can only be realized when it is fully absorbed into the workflow by the organization and its users [8]. By comparing EHR adoption and assimilation, Upadhyay and Hu [9] provided empirical evidence that organizational assimilation over adoption can significantly improve patient treatment efficiency. Thus, this study aimed to explore how EHRs contribute to a decrease in medical errors using the expanded DeLone and McLean (D&M) Information System (IS) Success Model as the theoretical framework.

Thousands of people seek medical attention every day from a small group of doctors working in public health to improve the health of communities. Patient health care services must be provided more quickly to cater for the health needs for these communities. There are many problems with the public health care system, including long patient wait times, poor health care service, and inadequate infrastructure. Communities report that services provided by facilities fall short of fundamental standards of care and patient expectations despite government efforts to improve the quality of health care services [10]. In South Africa, more than half of the public health care facilities keep their records on paper, even though the country has adopted several EHR systems [11]. According to Rumball-Smith et al [5], using EHR to manage patient documentation could improve health care services. Despite their importance, medical records are common to be mishandled, resulting in patient files having medical records missing and the inability to get the correct treatment. Misplaced or absent records may have a negative effect on patients’ quality of life [12]. It is currently acknowledged that modern productivity, efficiency, and effectiveness are necessary for the facilitation of medical care [13].

Losing a patient’s medical records would be the worst-case situation because it could result in more issues, a wrong diagnosis, or, in severe circumstances, the patient’s death [14]. In 1 case, where the woman gave birth to twins in the hospital, the Pietermaritzburg High Court ordered a KwaZulu-Natal district hospital to turn over medical information to the plaintiff’s attorney in July 2006. In addition, hospital incompetence is said to have resulted in the patient losing 1 of the twins, and the surviving twin developing cerebral palsy. Medical records may occasionally be difficult to locate in the filing department due to a variety of issues, including documents being misfiled or misplaced [15]. These records, which are subsequently discovered in their offices, are occasionally misplaced by medical personnel. Furthermore, although the patient’s status would have changed by the time the replacement record is found, the duplicate record would continue to exist.

This research developed a theoretical framework and a survey instrument consisting of questions to evaluate the efficacy of organizational EHR in day-to-day operations from the viewpoint of the nursing staff in residential adult care facilities. System quality, information quality (IQ), service quality (SQ), usability, user happiness, and net benefits were the 6 variables that made up the updated D&M IS Success Model that was part of the recommended study model.

### The DeLone & McLean IS Success Model

The D&M IS Success Model [16] has been 1 of the most popular measurement models in the IS industry when it comes to evaluating IS success. Numerous topics pertaining to the ongoing use of ISs have been investigated, with a focus on the model of IS success, which is theoretically based on DeLone and McLean’s [16] work. Studies [17,18] have indicated that the D&M model is a reasonably developed theoretical model that is frequently used to predict people’s behavior in a range of situations. Six interrelated IS success dimensions were identified using the original model. DeLone and McLean [16] proposed several factors that could be used to define IS success: system quality, output IQ, consumption (use) of the output, user response (user satisfaction), impact of the IS on user behavior (individual impact), and impact of the IS on organizational performance (organizational impact). This model indicated the causal and temporal relationships between the 6 characteristics and offered a system for categorizing the wide range of IS success measures. The D&M IS Success Model was updated 10 years later by DeLone and McLean, who combined organizational and individual impacts into a single impact variable known as “net benefit” and included SQ as a new dimension of measuring IS success [19]. The updated model [20] places strong emphasis on the value of gauging the effectiveness of IS variables.

The D&M IS Success Model [21] has been used to assess EHR implementations in numerous studies. It comprises elements of system quality, IQ, intention to use, satisfaction, actual use, and individual and organizational impacts. In the domain of health care, Jeyaraj [22] combined the Technology Acceptance Model (TAM) with the D&M IS Success Model. Consequently, he learned that having enough information available, having a well-designed interface, and having up-to-date information about the system are all crucial factors for an IS to succeed. The system’s design is the most crucial element since success is determined by these factors. Noushi and Bedos [23] discovered certain guidelines that need to be followed while creating a new IS that may be used for patient work and diagnostics in a dental clinic setting. Based on the reviewed literature, this study sought to offer a research model to determine the effects of EHR on better coordination of patient care (BCP) in public hospitals. In the context of an HIS in a low- to middle-income country, the study validated the updated D&M IS Success Model. Following is a description of the research model and the proposed research model’s hypotheses.

### Research Model

**Figure 1:**
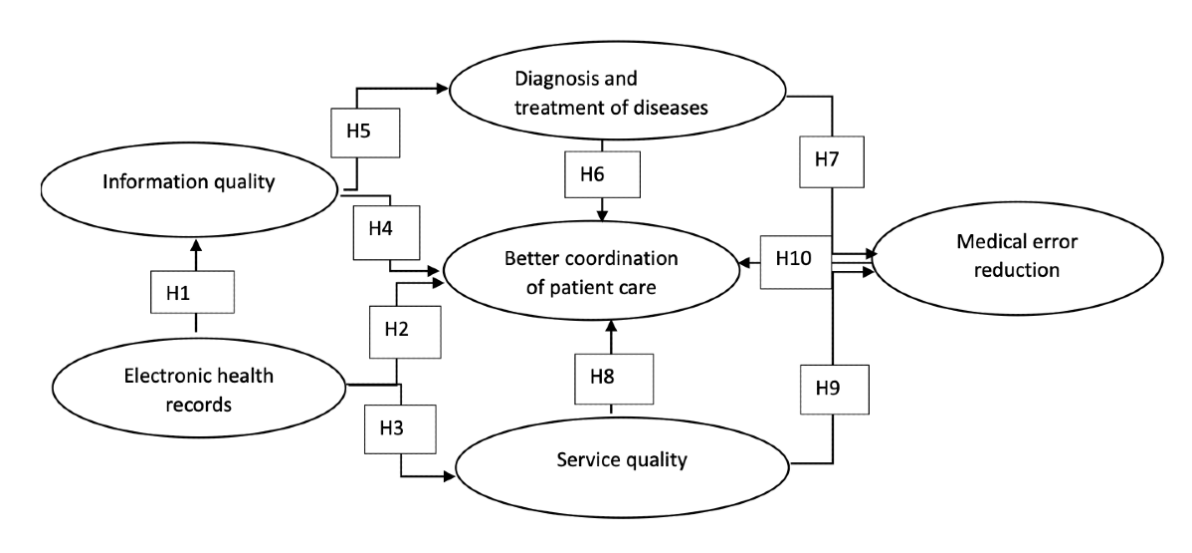
Research model. H: hypothesis.

The proposed research model is applied only to determine the effects of EHR on BCP in public hospitals. According to the objective of the study, the review of the literature, and the framework or TAM, the research model, as presented in Figure 1, adopted and incorporated the constructs of the D&M IS Success Model. A variable is defined as anything that has a quantity or quality that varies [24]. BCP was the dependent variable in this study’s research model, which consisted of 3 independent variables or predictor factors. The independent variables in the study were EHR, IQ, medical error reduction (MER), diagnosis and treatment of diseases (DTD), and SQ. The model constructs are described in Figure 1.

### Hypothesis Development

Based on Figure 1, the 3 independent or predictor factors in the research model were:

- EHR: Digital copies of comprehensive patient records kept by health care professionals, containing information such as patients’ medical history, diagnoses, and prescriptions
- IQ: The quality of information contained in the EHR system
- SQ: The quality of service provided by the health care facility, which can be influenced by factors such as the EHR system and the quality of information it contains

Every health care system aims to provide high-quality care [25]. Digital copies of thorough patient records that a health care professional keeps are called EHR or electronic medical records. According to Mitchell [26], EHR provide a comprehensive picture of a patient’s medical history, improving care coordination. This history includes information about past medical conditions, prescription medications, allergies, and test results. The goal of an EHR is to guarantee that the patient receives care that is both effective and efficient. EHRs improve efficiency and accuracy, while making patient medical records easier to access and share. Furthermore, it improves treatment quality by allowing clinicians to quickly communicate, analyze data, and identify trends [9]. The following hypotheses were presented to investigate these relationships:

- Hypothesis (H)1: EHR have a significant positive influence on IQ.
- H2: EHR have a positive significant influence on BCP.
- H3: EHR have a positive significant influence on SQ.
- H4: IQ has a significant positive influence on BCP.
- H5: IQ has a significant positive influence on DTD.

The accurate diagnosis provided by this crucial information helps save time and money [12]. Patient data from the EHR system can be coordinated across numerous organizations because they are universally available. It is easier to read data between different EHR systems, since standardization of data according to a common set of standards is encouraged [27]. This major advantage of electronic records probably boosts the effectiveness of all health systems worldwide. Another crucial issue that health care facilities must address is preventive care [9]. More preventive treatment is expected to significantly improve patient outcomes. EHR systems are an excellent tool for helping preventive care initiatives. The following hypotheses were presented to investigate these relationships:

- H6: DTD has a significant positive influence on BCP.
- H7: DTD has a significant positive influence on MER.

Furthermore, EHR systems provide medical practitioners with more data so they can create campaigns for preventive health care [9,28]. EHR systems improve operational effectiveness and reduce error rates, immediately enhancing the standard and safety of patient care. EHR frequently encourage collaboration between organizations and the development of more robust institutions. EHR systems encourage improved hospital-wide communication and collaboration. For health care professionals, the issue of medical errors as an element of SQ has become crucial. According to Fraser et al [29] the SQ technique helps understand patient expectations and facilitates changes in medical practices, increasing patient happiness and compliance, while also improving the quality of the medical services provided. The following hypotheses were presented to investigate these relationships:

- H8: SQ has a significant positive influence on BCP.
- H9: SQ has a significant positive influence on MER.
- H10: BCP has a significant positive influence on MER.

## Methods

### Research Design

The Dr George Mukhari Academic Hospital (DGMAH), a tertiary hospital located approximately 30 km north of Tshwane (Pretoria, South Africa), was the site of the study. Students in health sciences from the Sefako Makgatho Health Sciences University (SMU) use the DGMAH as a training hospital. It consists of 39 wards grouped together according to therapeutic specialties. A cross-sectional analytical research design was used for this investigation. Connelly [30] stated that all the data for a cross-sectional study should be gathered at once. Such studies are useful for recording the state of phenomena or the relationships between phenomena at a particular moment in time, since the phenomenon being studied is captured during a single data collection session. Data for the study were gathered at the DGMAH between August 2020 and July 2021. The sample size for this research was 300 medical health care professionals.

In this study, convenience sampling was the approach used in the participant selection process. Convenience samples are used in surveys where respondents are offered the choice to participate or not participate. This sampling is not probabilistic in any way. Probabilistic sampling involves selecting a sample using a probabilistic method without consulting the individuals selected [31]. Convenience sampling, according to Etikan et al [32], is a type of nonrandom sampling in which participants are selected from the target population only if they meet a specific set of pertinent practical requirements. Most often, convenience sampling is used to obtain information from subjects who are easy for the researcher to enroll into the study [32]. There are a number of inherent disadvantages to the convenience sampling technique. With this kind of sampling, biases in the sampling process and systematic errors could arise. In this sense, bias resulting from self-selection and noncoverage taints the convenience samples. Even though noncoverage is avoided and a sampling frame with a random pool of subjects is obtained, if empirical studies use nonprobability convenience samples, the researchers typically are unable to discharge self-selection, because individuals choose whether to complete the survey or participate in the interview at their own discretion. Furthermore, it is not possible to interpret the *P* value in a meaningful way. Alvi [33] further contended that the target population groups should be sufficiently inclusive to be further subdivided into an infinite number of categories that are relatively distinct from one another and therefore not representative of one another.

Teclaw et al [34] found that survey participants occasionally give up before completing the questionnaire. Therefore, it is imperative that the demographic information part of the survey be the first to be completed. Demographics are essential for comparison and descriptive reasons in any study. According to Teclaw et al [34], starting the survey with demographics enhances the item response rate. The goal of this research was to use EHR as a system to determine how IQ, MER, DTD, and SQ, which are independent variables, influence the dependent variable, BCP. Data were collected from the DGMAH, and the constructs were determined using the reviewed literature. Data were collected using a 5-point Likert scale (strongly disagree, disagree, neutral, agree, and strongly Agree). with items made up of demographic and background information, as well as D&M model variables. For the purposes of this study, the questionnaire items were adapted from conventional forms of the TAM, drawing on the relevant literature.

### Ethical Considerations

The study was approved by the University of South Africa–College of Agriculture and Environmental Sciences (UNISA-CAES) Health Research Committee (reference number 2019/CAES/075). The DGMAH, Office of the Director of Clinical Services, gave its approval for the study to be carried out at the hospital. Each participant provided signed informed consent, which included details about the researcher, the purpose of the survey, its length, privacy protection protocols, and other information. The study’s data were anonymized and deidentified.

### Multiple Linear Regression

The rationale behind the selection of multiple linear regression in this study was its ability to evaluate the relationships between a single continuous dependent variable and multiple independent variables. This fit well with the study’s goal of determining how different factors affect the reduction in medical errors. For that reason, multiple linear regression was used to investigate the relationship between the dependent variable (MER) and multiple independent variables (EHR, IQ, DTD, BCP, SQ). This method is appropriate when there is 1 continuous dependent variable hypothesized to be associated with 2 or more independent variables.

The specific variables included in the regression model were selected based on the research model and hypotheses developed from the literature review. The model aimed to test how EHR implementation influences MER, both directly and through potential mediating variables, such as IQ, DTD, SQ, and BCP.

Before conducting the regression, necessary assumption checks were performed:

- The Durbin-Watson statistic (2.119) indicated no significant autocorrelation in the residuals.
- The variance inflation factor (VIF) values were all below 5, suggesting no problematic multicollinearity.
- The histogram of standardized residuals followed a reasonably normal distribution.
- The Bartlett sphericity test (*P*<.05) and Kaiser-Meyer-Olkin (KMO) value (0.727>0.5) indicated sampling adequacy for factor analysis.

### Data Analysis

SPSS Statistics (IBM Corp) was used to perform data analysis. A reliability test on the entire data set resulted in a Cronbach α value of .94, which confirmed the reliability of the data for further analysis. Data analysis and results were divided into 3 sections to cover descriptive analysis, factor analysis, and multiple regression analysis. In addition, quantitative data were analyzed to determine the causal relationship between the independent variables (EHR, IQ, MER, SQ, DTD) and the dependent variable (BCP). Inferential analysis was conducted that involved examining the nature of relationships between the variables under study using the Pearson correlation coefficient. Correlation and regression analyses were used in the inferential analysis. The data analyzed were presented using tables, correlation, regression, and ANOVA.

## Results

### Demographic Information

Table 1 displays the demographic profile of the survey participants. The sample was made up of 89 (29.7%) males and 211 (70.3%) females. Approximately 34% (n=102) of the respondents were in the 31-40–year age range, while 8.7% (n=26) were younger than 25 years. In addition, 243 (81.4%) were nurses, and 57 (18.6%) were medical professionals. Furthermore, 23 (5.0%) had less than a year’s experience, 39 (13.0%) had 2-5 years’ experience, 162 (5.4%) had 6-10 years’ experience, and 76 (28.0%) had more than 10 years’ experience. The structural model was put to the test on 35 items using the Bartlett sphericity test and KMO sample adequacy. Kaiser [35] argued that a KMO value below 0.5 is insufficient. The KMO value for this study was 0.727, indicating that the sample was sufficient and that factor analysis could be carried out. Furthermore, the Bartlett sphericity test was performed, and the result of *P*<.05 indicated that there was a statistically significant association between the variables.

**Table 1:** Demographic information.

Demographics			Participants (N=300), n (%)
**Gender**			
	Male	89 (29.7)	
	Female	211 (70.3)	
**Age (years)**			
	<25	26 (8.7)	
	25-30	98 (32.6)	
	31-40	102 (34.0)	
	41-50	57 (19.0)	
	>50	17 (5.7)	
**Occupation**			
	Medical doctor	16 (5.0)	
	Pharmacist	12 (4.0)	
	Radiologist	10 (3.3)	
	Physiotherapist	9 (3.0)	
	Nurse	243 (81.4)	
	Dentist	10 (3.3)	
**Work experience (years)**			
	<1	23 (5.0)	
	2-5	39 (13.0)	
	6-10	162 (54.0)	
	>10	76 (28.0)	

### Reliability and Validity

To determine the internal consistency and relationship of the items on the scale, a reliability analysis was performed. Cronbach α was used to evaluate the dependability of 47 items. Cronbach α values exceeding 0.5 were considered as being within the acceptable range. The Cronbach α values of 7 variables were over 0.5 based on the indicated value, indicating strong consistency for those items. Items with values less than 0.5 were eliminated [36]. The reliability analysis of the 6 constructs is shown in Table 2.

**Table 2:** Reliability analysis.

Variable	Items, n	Cronbach α
DTD^a^	6	.783
BCP^b^	5	.852
MER^c^	5	.741
SQ^d^	5	.752
EHR^e^	5	.789
IQ^f^	5	.858

^a^DTD: diagnosis and treatment of diseases.

^b^BCP: better coordination of patient care.

^c^MER: medical error reduction.

^d^SQ: service quality.

^e^EHR: electronic health record.

^f^IQ: information quality.

### Multiple Linear Regression

Ten variables were subjected to multiple linear regression to measure the success of the structural model in determining the effects of EHR on the reduction in medical errors in DTD in public hospitals. This resulted in an *R*^2^ change, which showed an increase in variance accounted for by the new interaction term. The *R*^2^ change increased by 0.159, indicating a 15.9% increase in the amount of variation that the extra interaction term could explain. It is important to note that the increase in variation was statistically significant (*P*<.05), indicating that EHR, SQ, and IQ all significantly have a significant positive influence on MER. Table 3 outlines the results of ANOVA for IQ, DTD, BCP, SQ, and EHR as mediating variables of MER.

The relative importance of each construct was represented by the model’s standardized coefficients. The findings showed that there is no statistically significant relationship between MER and knowledge quality (KQ; β=.043, *t*=0.705, *P*<.05). MER and IQ had a negative and statistically insignificant relationship according to the predictor variables (β=–.080, *t*=–1.320, *P*<.05). However, there was a statistically significant relationship between MER and EHR (β=.125, *t*=2.043, *P*<.05). In general, the results showed a strong statistically significant correlation between the dependent variable MER and the predictors IQ, DTD, BCP, SQ, and EHR.

**Figure 2:**
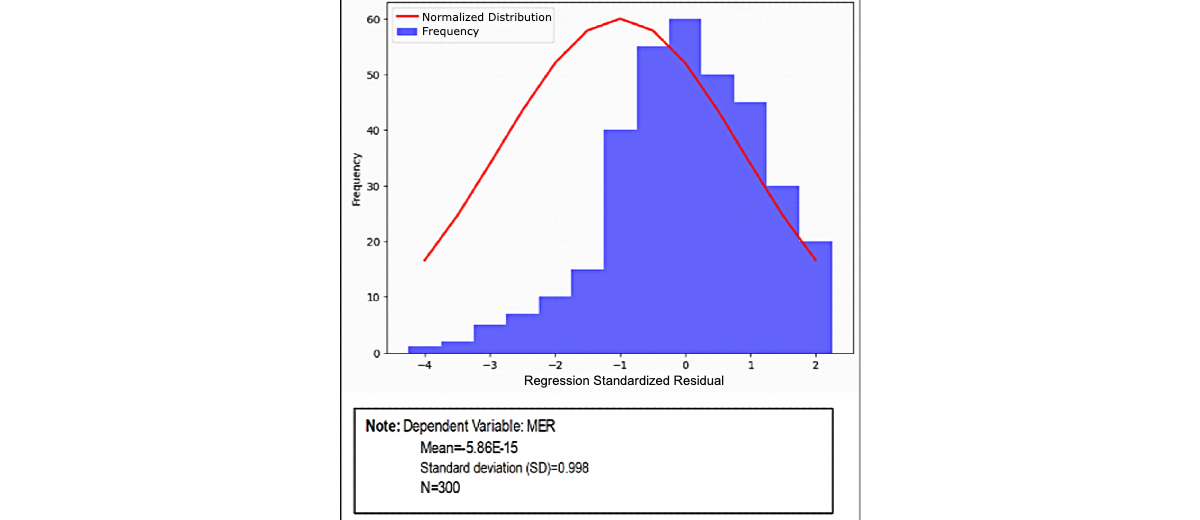
Histogram of standardized residuals for MER. MER: medical error reduction.

In this study, IQ, SQ, DTD, BCP, and EHR were all analyzed using hierarchical multiple regression. Table 4 represents the outcomes of the moderated regression analysis that are displayed in Figure 2, which shows the histogram of residuals in the regression model for the dependent variable, MER. The residual histogram was found to be reasonably normal and to be close to the normal curve.

**Table 3:** Summary of the regression model for analysis of success factors for model 1.^a^

Success factor		Value
*R*		0.399
*R^2^*		0.159
Adjusted *R*^2^ (SE)		0.154 (0.47074)
**Change statistics**		
	*R*^2^ change	0.159
	*F* change	28.055
	*df*1	2
	*df*2	295
	Significant *F* change	0
	Durbin-Watson autocorrelation	2.119

^a^Predictors: (constant), information quality (IQ), electronic health record (EHR), diagnosis and treatment of (DTD), service quality (SQ), and better coordination of patient care and (BCP); dependent variable: medical error reduction (MER).

**Table 4:** Regression coefficients of variables included in the optimized model.

Variable^a^	Unstandardized coefficient, B (SE)	Standardized coefficient, β	*t* test (*df*)	Significance	95% CI	Collinearity statistics	
						Tolerance	VIF^b^
(Constant)	3.903 (0.482)		8.100	0.000	2.954 to 4.851		
SQ^c^	0.035 (0.050)	.043	0.705 (0.600)	0.481	–0.064 to 0.135	0.976	1.024
IQ^d^	–0.061 (0.046)	–.080	–1.320 (–0.988)	0.188	–0.154 to 0.030	0.986	1.014
EHR^e^	0.136 (0.066)	.125	2.043 (2.890)	0.042	–0.005 to 0.067	0.975	1.026
BCP^f^	0.030 (0.207)	.016	0.144 (2.548)	0.085	–0.077 to 0.133	0.964	1.011
DTD^g^	0.181 (0.149)	.137	2.216 (3.118)	0.025	–0.112 to 0.171	0.971	1.028

^a^Dependent variable: medical error reduction (MER).

### Hypotheses

Multiple regression testing was performed to examine the effects of EHR on BCP in public hospitals, as well as to evaluate the statistical significance of each hypothesis. Of the 10 hypotheses, 6 (H1, H2, H4, H5, H7, and H10) were statistically significant in determining the effects of EHR on BCP in public hospitals according to the study’s findings, with *P*<.05. The results of the hypothesis testing are summarized in Table 5.

**Table 5:** Summary of the results of hypothesis testing.

Hypothesis (H)		Results	Outcome
H1	EHR^a^→IQ^b^	*P*=.028<.05, β=.354	Accepted
H2	EHR→BCP^c^	*P*=.010<.05, β=–.391	Accepted
H3	EHR→SQ^d^	*P*=.226>.05, β=.109	Rejected
H4	IQ→BCP	*P*=.010<.05, β=–.391	Accepted
H5	IQ→DTD^e^	*P*=.021<.05, β=.329	Accepted
H6	DTD→BCP	*P*=.229>.05, β=–.129	Rejected
H7	DTD→MER^f^	*P*=.002<.05, β=.415	Accepted
H8	SQ→BCP	*P*=.224>.05, β=.100	Rejected
H9	SQ→MER	*P*=.990>.05, β=.001	Rejected
H10	BCP→MER	*P*=.021<.05, β=.329	Accepted

^a^EHR: electronic health record.

^b^IQ: information quality.

^c^BCP: better coordination of patient care.

^d^SQ: service quality.

^e^DTD: diagnosis and treatment of diseases.

^f^MER: medical error reduction.

### Final Research Model

**Figure 3:**
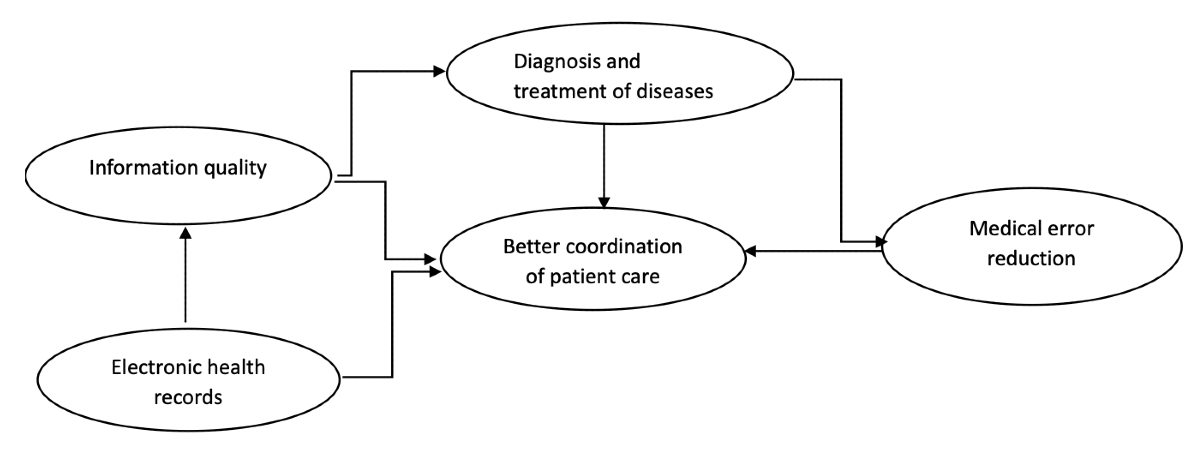
Revised research model.

The final proposed research model was reviewed by removing the rejected hypotheses that were not significant and keeping the variables supported by the accepted hypotheses based on the findings of the hypothesis testing and the significance level. These variables included MER rates, EHR, and IQ. Figure 3 shows the revised recommended model to examine the variables to determine the effects of EHR on BCP in public hospitals.

## Discussion

### Principal Findings

Developing a proposed model for reducing medical errors based on the updated D&M IS Success Model as the underpinning theory was the goal of this study. Empirical research was conducted on the suggested model, with medical professionals chosen from the DGMAH as participants. The 5 components of the suggested conceptual model were as follows: MER as the dependent variable, improved BCP, DTD, SQ, and EHR. Based on the validation procedure, the model was updated and changed. Of 10 hypotheses, 6 (H1, H2, H4, H5, H7, and H10) were statistically significant in predicting the effects of EHR on BCP in public hospitals according to the study’s results (*P*<.05). The use of EHRs was found to improve patient care coordination and IQ in a statistically significant way. EHR systems are intended to facilitate effective coordination of patient care by practitioners and support evidence-based decision-making [19]. The benefits of implementing EHRs, such as better patient outcomes, more patient safety measures, and lower costs, have also been noted in the literature [19].

The results of this study underline the notion that EHR-based clinical data offer several benefits over traditional medical records. As a result, they greatly improve overall health quality. They also become readily accessible through a range of communication channels [37]. This ensures that medical personnel treat patients correctly and greatly improves patient outcomes (H1, H2, H4). Additionally, the findings are consistent with those of previous studies [9], which indicated that EHR systems improve patient care quality and increase patient safety, specifically through gains in operational efficiency and a reduction in errors. Fraser et al [29] indicated that the EHR system also tends to promote stronger institutions and cooperation between organizations.

However, other studies have supported the findings of H5 and H7 by demonstrating that EHR systems encourage greater information exchange and interorganizational cooperation [38]. This outcome was in line with most of the earlier research [25]. Preventive treatment is considered crucial to improving patient outcomes and reducing medical expenditures for both individuals and the health system, particularly in regions that are susceptible to certain disease epidemics. The findings aligned with those of Tsai et al [39], who suggested that EHR systems can facilitate the creation of novel and evidence-based treatment objectives, in addition to enhancing data analytics and identifying strategies to improve patient outcomes. The study’s conclusions imply that by enhancing the capacity of health care organizations to communicate with patients, particularly those who require preventive care measures, the use of EHR systems can help improve preventive care. EHR in clinics can help medical professionals diagnose patients, treat them, and perform other tasks more accurately [29]. According to research by O’Donnell et al [40], physicians who are older and less tech savvy would likely be against the adoption of new technology. In this study, 5.6% of participants were older than 50 years and 19% were 41-50 years old. Therefore, it may be said that most of the participants were in the age range of 21-40 years. Based on the results, it appears that most participants were eager to use EHRs.

In supporting the findings of H10, Motsi and Chimbo [15] found that their survey results were in line with the conclusions, stating that the primary duty of all health care providers is to provide medical care. A well-known indicator of the effectiveness of hospital health services is patient satisfaction. Patient satisfaction is a critical indicator used to evaluate the quality of health care services rendered [25]. These results also support those of a study by Mohd and Chakravarty [41], who showed that patient-perspective evaluation of health service delivery has gained popularity and is now a fundamental component of all health systems because it serves as a useful gauge of service delivery effectiveness, especially in public health facilities. In this investigation, H3, H6, H8, and H9 were rejected. To further validate the results of the rejected hypotheses, more studies should be conducted in other public hospitals.

The insignificant relationship between SQ and BCP (H8 rejected) could potentially be explained by the fact that SQ encompasses many factors beyond just care coordination. Although high SQ is desirable, it may not directly lead to better coordination if other systemic issues exist. This highlights the need for a multifaceted approach targeting different aspects of health care delivery. The lack of a significant relationship between SQ and MER (H9 rejected) is somewhat surprising, as one would expect higher SQ to correspond with fewer errors. However, it underscores the fact that medical errors can stem from a complex interplay of factors, such as staffing levels, training, and communication protocols, rather than just SQ perceptions. Dedicated interventions focused on error prevention may be needed. The rejected H6 suggests that although accurate DTD is crucial, it alone may not automatically translate into BCP if there are disconnects in the overall continuum of care processes. Improving DTD must go hand in hand with enhancements in teamwork, information sharing, and smooth care transitions.

To effectively leverage the benefits of EHR for improving patient care coordination, a multipronged systems-based approach is recommended. This entails concurrently targeting electronic records, IQ, care coordination processes, accurate diagnosis/treatment, and error reduction through integrated efforts rather than siloed initiatives. Providing comprehensive training and clinical decision support tools can enable health care professionals to optimize EHR use for precise diagnosis, treatment planning, and error prevention. Clear protocols and accountability measures must be implemented to ensure seamless flow of information from EHR across the entire continuum of care, enabling truly coordinated services. Regular assessment of SQ from the patient’s perspective through surveys is crucial, and the feedback obtained should drive quality improvement initiatives. Interdisciplinary quality assurance teams should be established to conduct root cause analyses of medical errors and devise preventive strategies that go beyond just SQ aspects. Furthermore, updating health care policies and funding models to incentivize the adoption of integrated EHR systems and prioritize care coordination activities is vital for sustainable progress in this domain.

### Limitations

Although the results offer useful information for assessing the impact of EHR on BCP in public hospitals, the generalizability of the findings was hampered by the study’s use of a single academic hospital as its study unit. In this study, the effects of organizational factors were not considered. Even though the results are significant, it is imperative that the proposed framework be assessed in light of many theories in further studies, even if it only includes constructs from 1 model. Further research is required to determine their impact on EHRs and on BCP in public hospitals. Organizational culture, managerial support, and implementation readiness are additional variables that should be considered.

The researchers were unable to compare the findings of the study in private hospitals, as the sample only consisted of health care professionals working at a public hospital. A comparative analysis between public and private hospitals may shed light on how and the extent to which organizational and environmental factors influence the implementation of EHR adoption. The fact that a self-report questionnaire was used to gather data may also have limited the accuracy of the responses. Credibility concerns could have been raised by the health care professionals’ answers if they had an unclear understanding of EHR. Interviews ought to be used in future studies. The capacity to collect rich, thorough data; elicit and explain participant responses; customize the interview to the requirements of the research project; build rapport and trust with participants; and be careful when researching sensitive subjects are just a few of the benefits that come with conducting interviews.

### Conclusion

The purpose of this study was to determine how EHRs’ impact improves patient care coordination in public hospitals. The study proposed a model to determine the factors associated with improved patient care coordination. The study examined data collected from 300 health care professionals at the DGMAH using a cross-sectional analytical research design, and 6 of the 10 hypotheses were found to be supported by the data. The study’s findings indicate that EHR are statistically significant in 2 areas: better IQ and BCP. It was found that better DTD, as well as BCP, are significantly impacted by the quality of information. However, it was observed that improved patient care coordination has a positive and considerable influence on reducing medical errors but has no discernible effect on disease diagnosis and treatment. In terms of SQ, it was found that there is no correlation between decreased medical errors and BCP. The government should move more quickly to put policies into effect to increase the eagerness of medical personnel to practice.

In this study the D&M IS Success Model served as the underpinning theory for the development of a framework for MER influenced by the integration of EHR. To address the current issues of health care costs for treatment patients, this framework offers a solution that enables quick access to patient records for more coordinated, efficient care in low- to -middle-income countries, especially in Africa. In addition, this study’s findings will assist stakeholders in better understanding the importance behind the integration of the eHealth system with the full implementation of electronic records in South African public hospitals. This understanding will help the department of health and stakeholders to make informed decisions regarding the integration of eHealth with electronic records, which has been implemented at a snail’s pace. In addition, by expanding the body of knowledge, the study advances the field of academia in eHealth and health informatics. Furthermore, the study also contributes to the body of knowledge in both the fields of e-HIS governance.

## Abbreviations

BCP: better coordination of patient care
D&M: DeLone and McLean
DGMAH: Dr George Mukhari Academic Hospital
DTD: diagnosis and treatment of diseases
EHR: electronic health record
HIS: health information system
IQ: information quality
IS: information system
KMO: Kaiser-Meyer-Olkin
KQ: knowledge quality
MER: medical error reduction
SQ: service quality
VIF: variance inflation factor
